# PSMA-1-DOTA Potentially for Effective Targeted Radioligand Therapy of Prostate Cancer

**DOI:** 10.1007/s11307-025-02046-9

**Published:** 2025-09-02

**Authors:** Xinning Wang, Olga sergeeva, Maxim Sergeev, Lifang Zhang, Zoey Lockwood, Patrick Wojtylak, Riley Sangster, David Reichert, Marc Berridge, Wolfgang Weber, Zhenghong Lee, James P. Basilion

**Affiliations:** 1 Biomedical Engineering, Case Western Reserve University, Cleveland, OH USA; 2 Radiology, Case Western Reserve University, Cleveland, OH USA; 3 Chemistry, Case Western Reserve University, Cleveland, OH USA; 4 Nuclear Medicine, Radiology, University Hospitals Cleveland Medical Center, Cleveland, OH USA; 5 3D Imaging, LCC, Little Rock, AR USA; 6 Klinikum Rechts Der Isar, Nuklearmedizin, Technische Universität München (TUM), München, Germany

**Keywords:** Prostate cancer, PSMA, Targeted radioligand therapy, Dry mouth

## Abstract

**Purpose:**

While PSMA-targeted radioligand therapy (RLT) has shown remarkable efficacy for treating end-stage prostate cancer, the α-emitting RLT often results in severe salivary gland toxicity, limiting its use. Various strategies to mitigate this side effect have been attempted with limited success. Accordingly, this study introduced a new PSMA-targeting ligand with more favorable binding characteristics than the existing ligands.

**Procedures:**

The binding affinity of PSMA-1-DOTA to PSMA was compared with that of PSMA-11 and PSMA I&T. Comparison of uptake in the salivary glands, kidneys and PC3pip tumor cells in the xenograft mouse models between [^68^ Ga]Ga-PSMA-1-DOTA, [^68^ Ga]Ga-PSMA-11 and [^68^ Ga]Ga-PSMA I&T was conducted with microPET/CT within the same week. The same mouse models were treated with [^177^Lu]Lu-PSMA-1-DOTA or [^177^Lu]Lu-PSMA-617. A compassionate use PET imaging study on a patient with metastatic castration-resistant prostate cancer was performed using [^68^ Ga]Ga-PSMA-1-DOTA.

**Results:**

The binding affinity of PSMA-1-DOTA to PSMA was found to be approximately four times greater than other PSMA-targeted ligands. Imaging with microPET/CT revealed significantly lower kidney, uptake and little salivary and lacrimal gland uptake with [^68^ Ga]Ga-PSMA-1-DOTA compared to other PSMA-radioligands. Preclinical efficacy studies demonstrated that [^177^Lu]Lu-PSMA-1-DOTA inhibited tumor growth comparable to that with [^177^Lu]Lu-PSMA-617, suggesting its potential to enhance the therapeutic window of targeted RLT by avoiding damage to the salivary glands. The compassionate use PET imaging confirmed the reduced salivary gland uptake of [^68^ Ga]Ga-PSMA-1-DOTA in the patient, indicating its potential utility as a targeting agent for RLT with α- or β-emitting radionuclides in patients with PSMA-positive prostate cancer.

**Conclusion:**

PSMA-1-DOTA shows reduced uptake in salivary glands while effectively targeting PSMA-expressing tumors, thus potentially avoiding the side effects of xerostomia, and possibly moving PSMA-targeted RLT to a more frontline therapy for prostate cancer rather than the current use as a last resort.

**Supplementary Information:**

The online version contains supplementary material available at 10.1007/s11307-025-02046-9.

## Introduction

Prostate specific membrane antigen (PSMA)-targeted radioligand therapy (RLT) with α- or β-particle emitting radionuclides have shown extraordinary efficacy in the treatment of end-stage prostate cancer in patients who have exhausted all approved options [1]. Remarkably, some patients achieved complete biochemical (PSA) and imaging (PET) responses after a few rounds of PSMA-targeted RLT using a radioligand such as PSMA-617 chelated with an α-emitting radio-metal Actinium-225 (^225^Ac) [2]. In parallel, the same PSMA-targeting ligand, when chelated with a β-emitting radio-metal Lutetium-177 (^177^Lu), also yielded some tumor control [3]. Noticeably, targeted α-RLT elicited a response from some patients who did not respond to targeted β-RLT [1].

Severe salivary gland toxicity, *i.e.*, therapy-induced dry mouth (xerostomia), resulting from targeted RLT and causing an inability to talk or swallow among other intolerable discomforts, has become the dose-limiting toxic side effect for RLT [4]. Xerostomia is more severe with α-RLT occurring in 85% of the cases [5] compared to β-RLT [2, 6, 7]. In approximately 10% of α-RLT cases, xerostomia is so severe that patients opt out of this potentially life-saving therapy even though some patients had yielded complete responses [8]. Similarly, other PSMA-targeting peptide ligands such as PSMA-11 chelated with a γ-emitting radio-nuclide Gallium-68 (^68^ Ga) (Locametz) or DCFPyL conjugated with Fluorine-18 (^18^F) (Pylarify), which have been used for PET imaging, also displayed strong and sustained uptake in all salivary glands (parotid, submandibular, and sublingual) in almost all scans. Further, a longitudinal study with ^124^I-MIP-1095 also showed strong salivary gland uptake that was retained for at least 96 hours [9], indicating ligand binding and retention in the salivary glands. Targeted RLT-induced xerostomia has prevented RLT from becoming more widely used for earlier therapy of metastatic prostate cancer [10].

Radiotherapy-induced dry mouth is not new phenomenon. For patients with head and neck cancer who suffered xerostomia after external beam irradiation, strategies to reduce the burden of xerostomia include: sialagogue medications, saliva substitutes, submandibular gland transfer, hyperbaric oxygen, vitamins, acupuncture or associated treatments [11] and stem/progenitor cells-based approaches for salivary gland repair and regeneration [12–14]. Using medications such as pilocarpine, cevimeline, or amifostine have shown some improved xerostomia outcomes, but caused other toxicity; submandibular gland transfer seemed to be effective but required elective surgery, and thus may not always be appropriate or practical [11].

Other strategies to mitigate dry mouth caused by RLT agents have been tested, including medications, surgery, and injections documented in a recent review [15]. For radioligand therapy-induced dry mouth, approaches like Botox injections and external cooling have been tested but showed limited success [16, 17]. Combining radiolabeled and non-radioactive ligands can reduce uptake in glands but may also affect tumor treatment efficacy [18]. PSMA inhibitors like 2-PMPA reduce uptake in both glands and tumors, compromising cancer treatment [19, 20], however, the 2-PMPA prodrug, JHU-2545, shows promise in reducing gland uptake with less impact on tumors, and clinical evaluations are ongoing [21]. Monosodium glutamate (MSG) or folic polyglutamate tablets, has also been attempted to block entrance to RLT agents and was successful in mice [22]. However, a clinical trial comparing tomato juice containing MSG with tomato juice (placebo) turned out to be negative [23]. In a Phase II clinical study, folic polyglutamate tablets were given and patients were instructed to hold them longer in the mouth to increase buccal absorption for better local protection [24]. The full impact on the severity of xerostomia as well as the safety profile of polyglutamate needs to be further investigated. The lack of impact of all these efforts underscores the persistent unmet need to reduce RLT uptake and retention in salivary glands.

**Fig. 1:**
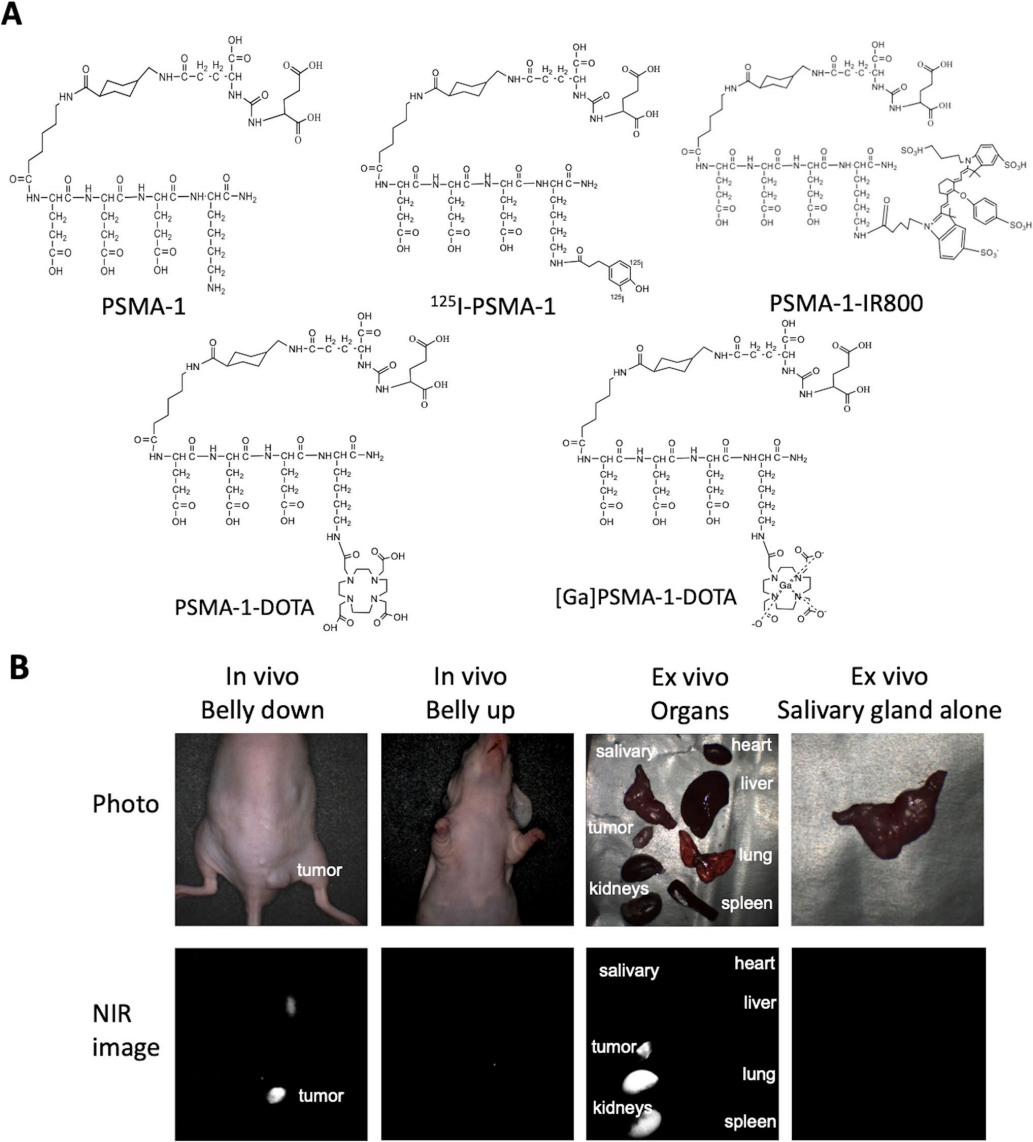
**A** Structures of rationally designed new PSMA ligands. PSMA-1 (far left) was developed by our group using rational design, and then labeled with ^125^I (^125^I-PSMA-1), IR800 for fluorescence imaging (PSMA-1-IR800), or ^68^ Ga via DOTA ([^68^ Ga]Ga-PSMA-1-DOTA) for nuclear scans. **B** Lack of Mouse Salivary Gland Uptake of PSMA-1-IR800. PSMA-1 was conjugated to IR800 near infrared dye and injected into mice harboring PSMA-expressing human tumors (PC3pip tumors). After 24 h the animals were imaged and sacrificed. The organs and tumors were removed for ex vivo imaging. PSMA-1-IR800 was only detected in the PC3pip tumor and kidney. No fluorescence was detected in the salivary glands or other organs including: heart, liver, lung, and spleen. Pictures are representative images of 3 mice

Using rationale design to improve selectivity, we have developed a new PSMA-targeting ligand, PSMA-1, that when conjugated with I-125 displayed a considerably lower uptake in the salivary glands while still effectively targeting PSMA-expressing tumors [25]. This raised the potential to avoid the intolerable side effects of xerostomia while maintaining tumor control, and possibly moving PSMA-targeted RLT from a last resort to a more available radiotherapy for prostate cancer. In this report, we summarized our further development of PSMA-1 with a DOTA chelate (PSMA-1-DOTA, Fig. 1A) for PET imaging and targeted RLT in animal models as well as a “first-in-human” scan with ^68^ Ga-PSMA-1-DOTA.

## Materials and Methods

### Probes Synthesis for Animal Use

*Synthesis of precursor PSMA-1-DOTA*: PSMA-1-DOTA was synthesized on solid phase by Fmoc-chemistry as reported previously at 0.1 mmol scale [26]. After Glu-CO-Glu’-Amc-Ahx-dGlu-dGlu-dGlu-Lys(Mtt)-rink amide resin was built on the resin, the Mtt group on Glu-CO-Glu’-Amc-Ahx-dGlu-dGlu-dGlu-Lys(Mtt)-rink amide resin was removed by 2% TFA in dichloromethane. The resin was washed with 2% DIPEA in DMF and resuspend in DMF, then 3 equivalents of DOTA NHS ester (Macrocyclics) and 3 equivalents of DIPEA was added. After the reaction was completed as indicated by Kaiser test (AAPPTEC), PSMA-1-DOTA was cleaved from the resin by trifluoroacetic acid (TFA)/water/triisopropylsilane (950:25:25) and purified by preparative high performance liquid chromatography (HPLC) using Luna 5 µ C18(2) 100 column (250 mm × 10 mm × 5 μm, Phenomenex, Torrance, CA) at a flow rate of 2.5 ml/min. The gradient used was 10%—40% acetonitrile against 0.1% trifluoroacetic acid over 30 min. Purity: > 98%. Retention time: 18.9 min (Figure S1A). EI-MS and MALDI-TOF–MS: C_62_H_100_N_14_O_27_, calculated: 1474.69; found: 1474.38 (Figure S1B-C). ^1^H NMR (500 MHz, D_2_O): δ (ppm)m 0.96 (m, 2H), 1.33 (m, 2H), 1.41 (m, 4H), 1.48 (m, 4H), 1.50 (m, 1H), 1.60 (m, 2H), 1.82 (m, 6H), 1.97 (m, 6H), 2.16 (m, 7H), 2.32 (t, 2H), 2.39 (t, 2H), 2.50 (m, 8H), 3.05 (m, 2H), 3.16 (t, 4H), 3.33 (br, 16H), 3.80 (br, 8H), 4.18 (m, 1H), 4.26 (m, 4H), 4.38 (m, 1H) (Figure S1D-F).

*Synthesis of cold Ga-PSMA-1-DOTA*: PSMA-1-DOTA (1.4 mg, 0.97 μmol) was dissolved in 400 μL of 0.2 M sodium acetate (pH 3–4), to which fivefold excess gallium nitrate (1.25 mg, 4.9 μmol) in 400 μL of 0.2 M sodium acetate (pH 3–4) was added. The reaction mixture was stirred at 90 °C for 1 h and went through HPLC for purification. The HPLC condition was the same as the purification of PSMA-1-DOTA. Yield: 1.1 mg, 78%. Purity: > 98%. Retention time: 18.7 min. EI-MS and MALDI-TOF–MS: C_62_H_98_GaN_14_O_27_, calculated: 1540.60; found: 1541.61 (Figure S2).

*Gallium-68 radiolabeling*: The synthesis of [^68^ Ga]Ga-PSMA-1-DOTA was carried out based on a protocol described by Scintomics GmbH for routine production of [^68^ Ga]Ga-PSMA-11. This protocol was improved and validated at University Hospital (UH) PET radiopharmacy, and generator elution mode was set up in accordance with Eckert & Ziegler directives for IGG-100 generator elution. The entire synthesis, including elution of radioactive gallium from the generator with 0.1 M hydrochloric acid, conjugation with PSMA-1 peptide, purification and formulation of the final product are automated by Scintomics GRP® synthesizer and controlled by the software using GMP-manufactured single-use sterile cassettes. Gallium-68 was produced at University Hospital (UH) PET Radiochemistry facility using a 50 mCi IGG100-50 M ^68^Ge/^68^ Ga generator purchased from Eckert & Ziegler Isotope Products. After the program started, the C^18^ cartridge was preconditioned with ethanol (5 mL) and washed with water (20 mL). The ^68^ Ga was eluted from ^68^Ge/^68^ Ga generator with 5 mL of hydrochloric acid (0.1 M) and the eluent was diluted with 6 mL water in the syringe. This solution was passed through the strong cationic exchange cartridge in proton cycle (PS-H^+^) where ^68^ Ga was retained, and subsequently eluted in the next step with sodium chloride solution (5 M, 1.7 mL) into the pre-heated reactor containing 20 µg PSMA-1 precursor peptide (added as 20 µL of stock solution with concentration of 1 mg/mL) and HEPES buffer (1.5 M, pH 4.5, 1.5 mL). The labeling took 5 min at 90 °C (temperature set for the heater). After labelling step, the reactor content was transferred onto the C^18^ Plus Light SPE-cartridge, where the labeled [^68^ Ga]Ga-PSMA-1-DOTA was retained while the ionic ^68^ Ga, HEPES, unreacted precursor and salts are washed out with water for injection (8 mL). The labeled [^68^ Ga]Ga-PSMA-1-DOTA product was eluted from the C^18^ cartridge with mixture of water for injection/ethanol (1/1, v/v, ~ 2 mL) through the 0.22 µm membrane sterile filter into the final product vial. Finally, the product is diluted with ~ 13 mL PBS through the same sterile 0.22 µm membrane filter into the final product vial. Samples were then aseptically removed for quality control testing. Radio-TLC and Radio-HPLC were performed using the same analysis for [^68^ Ga]Ga-PSMA-11 to confirm the success of radiolabeling. Similarly, [^68^ Ga]Ga-PSMA I&T was also synthesized using the Scintomics system for comparison scans. The products were diluted with ~ 13 mL sterile PBS in the final product vial. Samples were then aseptically removed for quality control testing.

*Lutetium-177 radiolabeling*: A stock solution of ^177^Lu (Eckert & Ziegler) in 0.04 M HCl was prepared in a clean reaction vial. The 177Lu was then diluted with up to 250 µL 0.25 M sodium acetate, pH 5.5. A solution of 25–250 ug PSMA-1-DOTA (1 mg/mL in 0.25 M sodium acetate, pH 5.5) was then added to the ^177^Lu solution. The reaction was allowed to mix for 60 min at 90 ˚C. The reaction was checked via radio-TLC and if the crude yield was below 90%, then an additional 25 µg PSMA-1-DOTA (1 mg/mL in 0.25 M sodium acetate, pH 5.5) was added with mixing continued for an additional 30 min. The crude reaction was then sterile filtered using a 0.22 µm sterile filter.

The product was analyzed by Radio-TLC on Macherey–Nagel silica gel 60 plates eluted with 100 mM EDTA in 100 mM PBS, pH 7.2 (Rf: [^177^Lu]Lu-PSMA-1-DOTA 0.0, ^177^Lu 0.6) and by RP-HPLC with a method developed using an Agilent 1090 HPLC equipped with a radiochemical detector. Specifically, the sample was analyzed on a Phenomenex Luna Omega C18 5 µm column with linear gradient of 100% of solvent A to 100% solvent B in 17 min. Solvent A is 0.1% TFA in water and solvent B is 0.1% TFA in acetonitrile. [^177^Lu]Lu-DOTA-PSMA-1 has a retention time of 11.5 min. Free ^177^Lu has a retention time of 4.5 min, Fig. 7S.

### Cell Lines

Retrovirally transformed PSMA positive PC3pip cells were obtained from Dr. Michel Sadelain in 2000 (Laboratory of Gene Transfer and Gene Expression, Gene Transfer and Somatic Cell Engineering Facility, Memorial-Sloan Kettering Cancer Center, New York, NY). The cells were last checked and authenticated by flow sorting and western blot in 2023. Cells were maintained in RPMI 1640 medium with 10% fetal bovine serum (FBS) at 37 °C and 5% CO_2_ under a humidified atmosphere.

### Competition Binding Assay

Competition binding assay was performed as described previously using N-[N-[(*S*)−1,3-dicarboxypropyl]carbamoyl]-*S*-[^3^H]-methyl-L-cysteine (^3^H-ZJ24) (GE Healthcare Life Sciences, Pittsburg, PA) [26, 27]. (S)−2-(3-((S)−5-amino-1-carboxypentyl)ureido)pentanedioic acid (ZJ24) was custom made by Bachem Bioscience Inc. (Torrance, CA). PSMA-11 and PSMA-I&T was purchased from MedChemExpress. PC3pip cells (5 × 10^5^) were incubated with different concentrations of PSMA ligands in the presence of 12 nM ^3^H-ZJ24 in 50 mM Tris buffer (pH 7.5) for 1 h. The cells were then washed 3 times with cold phosphate-buffered saline (PBS) and the radioactivity retained by cells was measured by scintillation counting. The concentration required to inhibit 50% of binding (IC_50_) was determined by GraphPad Prism 3.0

### Mouse Subcutaneous Xenograft Model

All animal procedures were performed according to Case Western Reserve University’s Institutional Animal Care and Use Committee (IACUA) approved protocols. Male athymic nude (Foxn1/nu/Foxn1/nu) mice 6–8 weeks old (Jackson Laboratory) were used to generate tumor xenografts. Upon arrival, mice were maintained in a 12-h light/12 -hour dark cycle with free access of water and food. PC3pip cells (1 × 10^6^) were suspended in 100 μL of Matrigel/PBS mixture (1:1) and injected to the right flank of the mice. Mice were then observed every other day and were ready to use when the tumor size reached about 100 mm^3^.

### Fluorescence Imaging with PSMA-1-IR800

PSMA-1-IR800 was synthesized as previously reported [26]. Male athymic nude mice bearing PC3pip tumor were injected with 2 nmol of PSMA-1-IR800 via tale vein injection. Mice were imaged at 24-h post injection using a Curadel RP1 camera using the 800 nm channel. After in vivo imaging, mice were sacrificed and organs such as salivary gland, heart, liver, lung, spleen, kidneys and tumor were excised for ex vivo imaging.

### MicroPET/CT imaging with ^68^ Ga-PSMA Agents

Male athymic nude mice (Foxn1/nu/Foxn1/nu, Jackson Laboratory) with or without subcutaneous PC3pip tumors were administered 7.4 MBq (200 μCi) of [^68^ Ga]Ga-PSMA-1-DOTA through tail vein injection. Five minute-static microPET scans were performed at 30 min and 1 h post injection using a Siemens Inveon microPET-CT scanner (Siemens Medical Solutions, Knoxville, TN). PET images were reconstructed using the default iterative reconstruction supplied by the vendor of Inveon. During imaging mice were under anesthesia with 2.0% isoflurane delivered in oxygen gas with a nose cone. Immediately after the micro-PET scan was complete, a CT scan was performed for better anatomic localization. Within a week, two other imaging sessions were carried out in 24- or 48-h intervals with the same mice injected with 7.4 MBq (200 μCi) of either [^68^ Ga]Ga-PSMA-11 or [^68^ Ga]Ga-PSMA I&T in 200 μL saline solution and similar microPET/CT scans were performed at 1 h post injection.

Regions of interests (ROIs) were drawn over PET/CT overlapped images to determine uptake in the parotid glands (left and right), lacrimal glands (left and right), kidneys (left and right) and PSMA-positive PC3pip xenograft tumors as well as muscle tissues (background). Standardized uptake values (SUVs, normalized by body weight) were calculated for quantification [28, 29]. Based on SUVmax values, tumor-to-organ ratios were calculated for the kidneys, salivary glands and muscle background for both [^68^ Ga]Ga-PSMA-11 and [^68^ Ga]Ga-PSMA-1-DOTA and are reported in Table 1.

**Table 1:** Tumor-to-Organ ratios comparing [68 Ga]Ga-PDMA-11 (PSMA-11) and [68 Ga]Ga-PSMA-1-DOTA (PSMA-1-DOTA)

(30 min)	PSMA-11	stdev	PSMA-1DOTA	stdev	(1 h)	PSMA-11	stdev	PSMA-1DOTA	stdev
kidney (L)	0.28254999	0.07309635	1.264329806	0.19923998		0.2862779	0.15425123	1.275128294	0.5964534
kidney (R)	0.24386086	0.06330612	0.466399815	0.53047377		0.2828353	0.17255101	1.064801961	0.78224407
salivary (L)	8.32085167	2.65596861	13.44835774	3.49398437		9.5732761	5.7184122	27.42536979	24.8643599
salivary (R)	7.54874766	1.35512259	13.60908704	4.04472816		8.8346404	4.89238421	27.03918133	24.6887017
muscle	18.4088062	6.21909533	18.67923866	3.68987496		31.645926	24.491019	42.9180275	43.1719857

1) The same animals (n=4) were scanned twice, PSMA-11 and then PSMA-1-DOTA, within a week;

2) The comparison was based on maximum SVUs (SUVmax) measured at 30 min and 1-hr post-injection;

3) L: left, R: right;

4) The salivary glands are the mouse parotid glands

### Tumor Inhibitory Activity of [^177^Lu]Lu-PSMA-1-DOTA

For treatment experiments, [^177^Lu]Lu-PSMA-1-DOTA was labeled by 3D Imaging, LCC with clinical grade [^177^Lu]Lu-PSMA-617 (Pluvicto, Novartis) used as positive control for RLT. Male athymic nude mice (Foxn1/nu/Foxn1/nu, Jackson Laboratory) with subcutaneous PC3pip tumors approximately 100 mm^3^ in size were randomly assigned to three groups of three mice each. On day 0, the mice were injected intravenously with either vehicle control (negative control) or received a single dose of 111 MBq (3 mCi) of [^177^Lu]Lu-PSMA-617 as a positive control. The third group of animals received a total of 111 MBq (3 mCi) of [^177^Lu]Lu-PSMA-1-DOTA administered in 4 doses (27.75 MBq each over 2 days). The second radioligand treatment was administered on day 13 with mice receiving a single dose of 111 MBq (3 mCi) for [^177^Lu]Lu-PSMA-1-DOTA and [^177^Lu]Lu-PSMA-617. Tumor volumes were measured every 3 to 4 days for 24 days following treatment.

### Toxicity Studies of PSMA-1-DOTA

After synthesis and purification of PSMA-1-DOTA for compassionate human use, good laboratory practice (GLP) toxicity studies were performed by ATRC Aurigon Toxicological Research Center Ltd. (Hungary). Eight-week-old male NMRI mice (Charles River Laboratories) were used for the study. The study was a single dose extended study designed to assess immediate effects of PSMA-1-DOTA injection (main animals) and recovery from immediate effects after 15 days (recovery animals). The main animal group had 20 mice, in which, 10 mice received 0.9% normal saline as controls, and the other 10 mice received 1.4 mg/kg of PSMA-1-DOTA via intravenous (i.v.) injection. In the recovery animal group, five mice received 0.9% normal saline, and the remaining five received 1.4 mg/kg of PSMA-1-DOTA intravenously. The dose level was based on micro-dosing used on humans and was 1000X the maximum human micro-dose of 100 ug per dose assuming an average human body of 70 kg. After injection (Day 1) blood sampling was performed on Day 2 for main animals (20 animals) and on Day 15 for recovery animals (10 animals). Recovery animals were observed for a 15-day period. Necropsy was performed on day 2 for main animals and day 15 for recovery animals. Clinical chemistry and histopathology studies were performed.

### “First in Human” Compassionate Use

[^68^ Ga]Ga-PSMA-1-DOTA was administered to a patient on a compassionate use basis. The production of [^68^ Ga]Ga-PSMA-1-DOTA was in compliance with the German Arzneimittegesetz (AMG), §13 2b, and the responsible regulatory authority (Government of Upper Bavaria) was duly notified. That is, [^68^ Ga]Ga-PSMA-1-DOTA was synthesized as described above at 0.4 mmol scale and the purity was confirmed by HPLC to be more than 98%. Written informed consent was obtained from the patient. 282 MBq (7.62 mCi) of [^68^ Ga]Ga-PSMA-1-DOTA was injected *i.v*. and a whole-body PET scan using a Siemens Biograph Vision with 2-min/bed from the top of the head to the thigh was taken at 1 h post-injection. PET images were reconstructed using the iterative OSEM with Time-of-Flight and resolution recovery ("TrueX") on the Biograph Vision system.

## Results

### Synthesis and Identity Confirmation

**Fig. 2:**
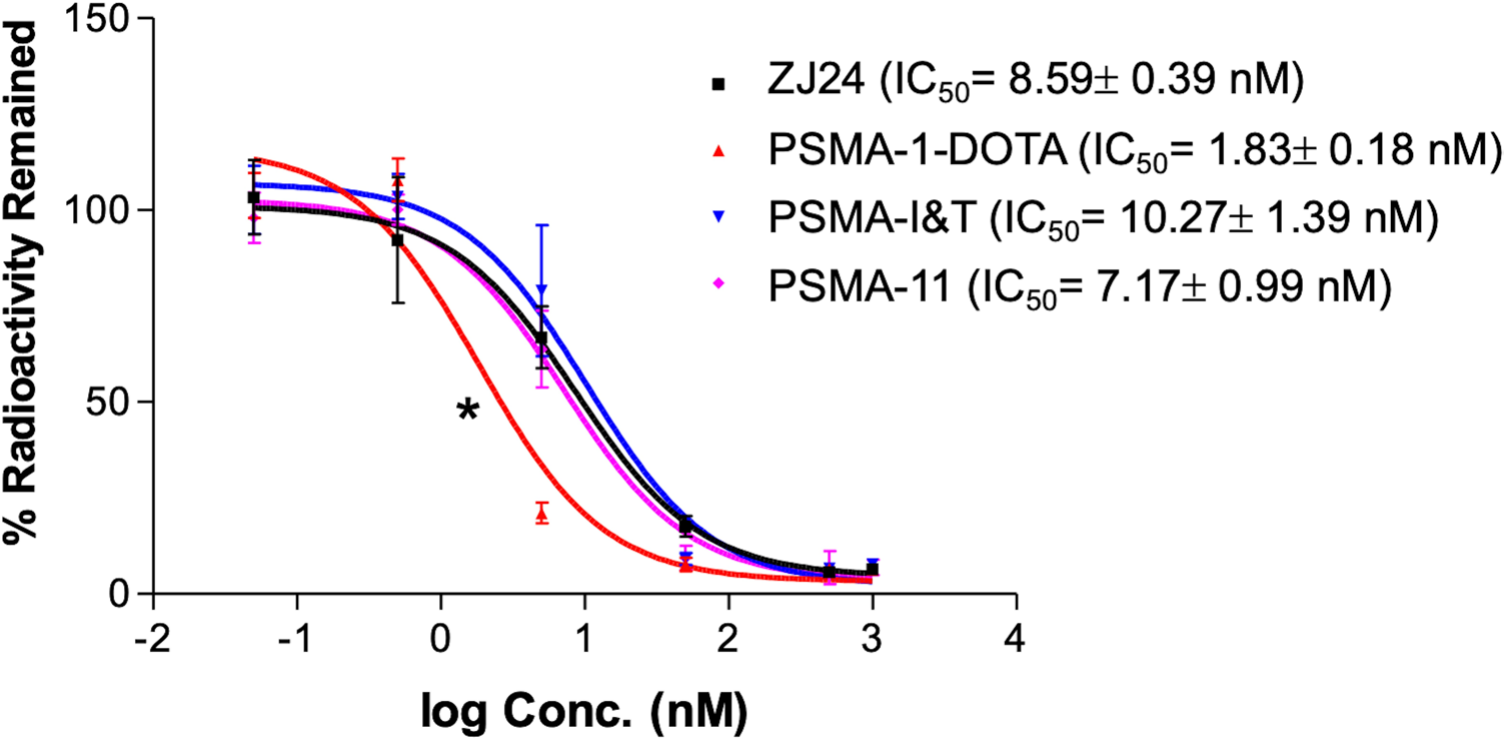
Higher affinity of PSMA-1-DOTA for PSMA. PSMA-1-DOTA compared to other PSMA-ligands using PC3pip cells through a competition binding assay against [^3^H]-ZJ24. Values are mean ± SD of 3 replicates. *: p < 0.001, PSMA-1-DOTA vs ZJ24, PSMA I&T and PSMA-11

Our distinct ligand design featuring sequential D-glutamate residues, PSMA-1 [26], (Fig. 1) has resulted in significant tumor uptake with little to no salivary gland uptake when labeled with ^125^I^25^, or IR800 [26], Fig. 1B. PSMA-1 was conjugated with the macrocyclic chelator DOTA resulting in PSMA-1-DOTA, Fig. 1A, as described in methods. The binding affinity of PSMA-1-DOTA was determined by radioactive competition assays against [^3^H]-ZJ24 and compared with other PSMA-targeted PSMA ligands, Fig. 2. Compared to PSMA-I&T, which is in clinical trials, and the FDA approved PSMA-11, in vitro the affinity of PSMA-1-DOTA was determined to be approximately fourfold greater using prostate cancer cells overexpressing the PSMA receptor (PC3pip cells) (p < 0.001). The ^68^ Ga-PSMA agents were synthesized with similar specific activity as [^68^ Ga]Ga-PSMA-11 from the same Scintomics module using a similar program with a high radiochemical purity (RCP) > 98% and a typical decay corrected chemical yield of ~ 55% (~ 45% non-decay corrected at EOS). The identity of [^68^ Ga]Ga-PSMA-1-DOTA was verified by HPLC (Figure S3) in two ways: first by spiking with the cold standard, resulting in co-elution (Figure S3A), and second by comparing the HPLC traces for the precursor (PSMA-1-DOTA) and [^68^ Ga]Ga-PSMA-1-DOTA (Figure S3B**)**. Radio-TLC displayed a clean peak for radiolabeled-peptide (Figure S3C). PSMA-1-DOTA also was determined to be stable for at least 1 week when incubated at 37 °C with either PBS, mouse or human serum (Figure S4).

### MicroPET/CT Scans

**Fig. 3:**
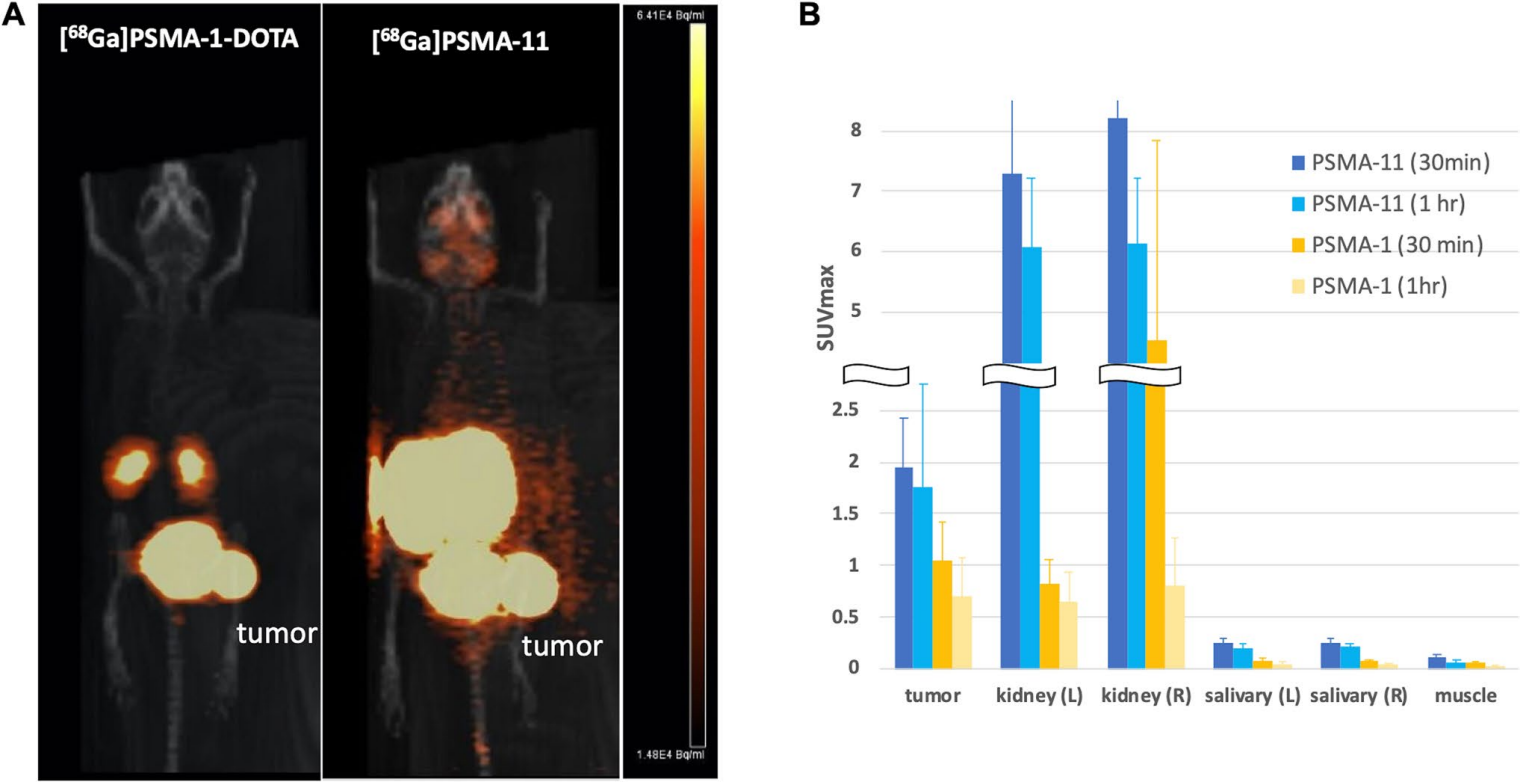
Micro PET/CT scan on mice bearing PC3pip tumor. **A** Comparison of micro-PET/CT images of [^68^ Ga]Ga-PSMA-1-DOTA and [^68^ Ga]PSMA-11 at 1 h post injection. Images showed lack of [^68^ Ga]Ga-PSMA-1-DOTA uptake in the salivary/lacrimal glands compared to [^68^ Ga]Ga-PSMA-11. The microPET/CT was performed one hour after injection of each radioligand. **B** Quantitative analysis of micro PET/CT images. Values are mean ± SD of 4 mice

Both [^68^ Ga]Ga-PSMA-11 and [^68^ Ga]Ga-PSMA-1-DOTA were used for microPET/CT scans to quantify tumor and salivary gland uptake. To avoid experimental variations due to tumor differences, comparison of the uptake of [^68^ Ga]Ga-PSMA-1-DOTA to that of [^68^ Ga]Ga-PSMA-11 was performed in the same tumor-bearing animals after clearance of [^68^ Ga]Ga-PSMA-1-DOTA. Animals bearing PC3pip tumor (n = 4) received 7.4 MBq (200 μCi) of [^68^ Ga]Ga-PSMA-1-DOTA, were scanned, and then rested for 24 h. After resting the animals were injected with an equal amount of radioactive [^68^ Ga]Ga-PSMA-11 and again scanned. Figure 3A shows fused microPET/CT images in which the PET images are displayed as a maximal intensity projection (MIP). Interestingly, ^68^ Ga-PSMA-1-DOTA identified the tumor comparably to [^68^ Ga]Ga-PSMA-11, but had significantly lower kidney uptake and no detectable salivary and lacrimal gland uptake at 1 h post-injection. Figure 3B provides quantification of the data obtained from microPET/CT scan of multiple animals showing almost 90% reduction in renal and salivary uptake with [^68^ Ga]Ga-PSMA-1-DOTA compared to [^68^ Ga]Ga-PSMA-11 but only approximately 50% reduction in tumor uptake at 1 h post-injection. Tumor-to-Organ ratios were calculated for the kidneys and salivary glands for [^68^ Ga[Ga-PSMA-11 and [^68^ Ga]Ga-PSMA-1-DOTA and tubulated in Table 1, further illustrating a higher tumor-to-kidney ratio and an even higher tumor-to-salivary ratio for [^68^ Ga[Ga-PSMA-1-DOTA vs. [^68^ Ga[Ga-PSMA-11. To avoid the"tumor sink"effect [30], where large tumors absorb most of the radioligand leaving normal tissues with lower uptake, [^68^ Ga]Ga-PSMA-11 and [^68^ Ga]Ga-PSMA-1-DOTA were also compared in mice without PC3pip prostate tumors. As shown in Figure S5, [^68^ Ga]Ga-PSMA-11 had significant uptake in the salivary glands. In contrast, none was noted for [^68^ Ga]Ga-PSMA-1-DOTA.

Tumor and salivary gland uptake of [^68^ Ga]Ga-PSMA-1-DOTA was also compared to [^68^ Ga]Ga-PSMA I&T uptake. The same mice were sequentially injected with [^68^ Ga]Ga-PSMA-1-DOTA, [^68^ Ga]Ga-PSMA I&T or [^68^ Ga]Ga-PSMA-11 following a clearance period of 24 h after each injection of each molecule. [^68^ Ga]Ga-PSMA-1-DOTA again showed significantly reduced salivary gland uptake as compared to the other two radioligands (Figure S6). The tumor and salivary gland uptake was measured using SUVs for each of the 3 probes.

### Tumor Inhibitory Activity of [^177^Lu]Lu-PSMA-1-DOTA

**Fig. 4:**
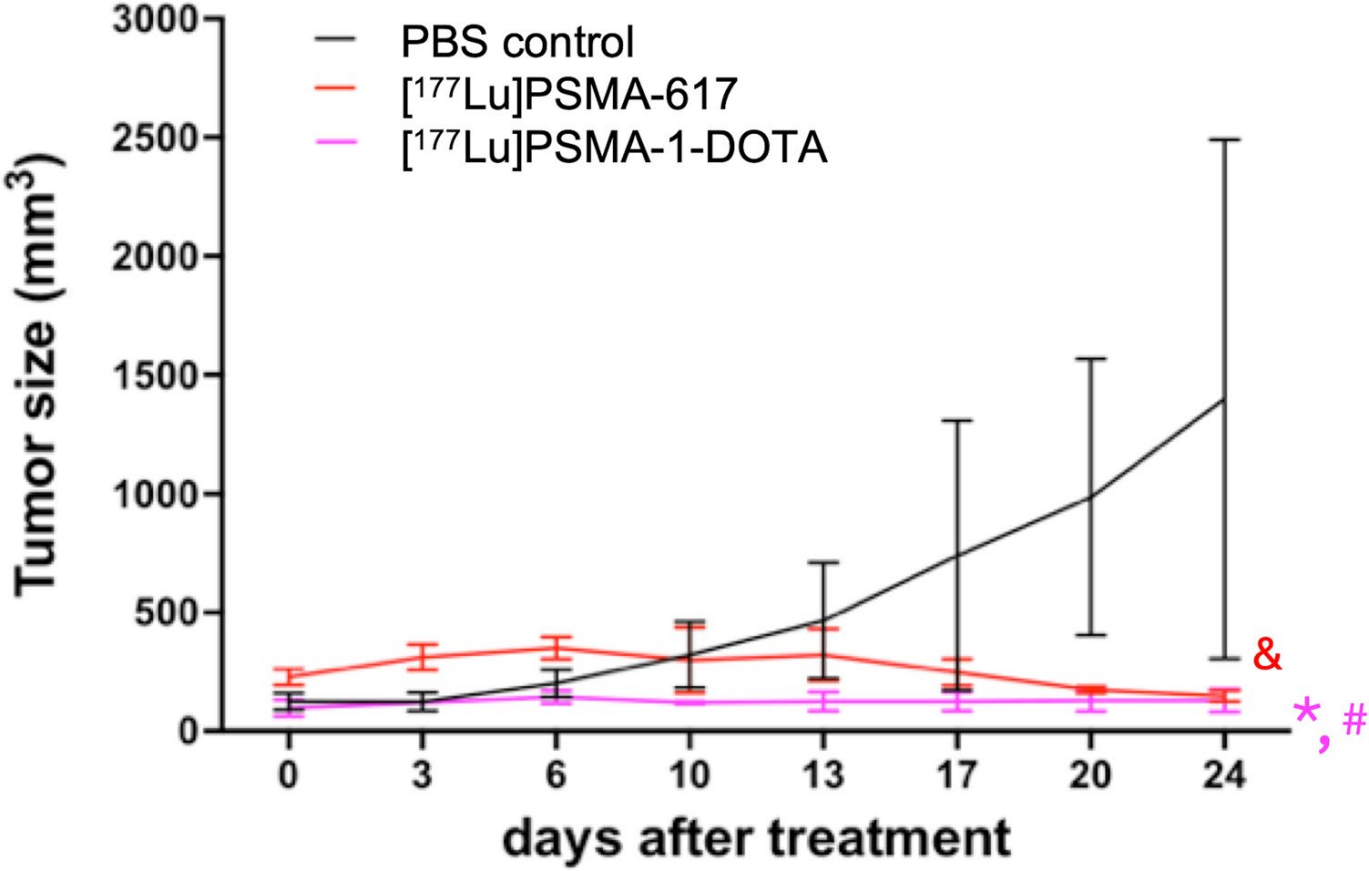
Efficacy of RLT for [^177^Lu]Lu-PSMA-1-DOTA, or [^177^Lu]Lu-PSMA-617. Animals bearing PC3pip tumors were administered IV PBS (control), [^177^Lu]Lu-PSMA-1-DOTA, or [^177^Lu]Lu-PSMA-617 on day 0 and 13 after tumors had reach 100 mm^3^. Tumor growth was measured using caliper measurements every 3–4 days. N = 6 in PBS control group and N = 3 in the two treatment groups. (#: p = 0.0057, [^177^Lu]Lu-PSMA-617 vs PBS. *: p = 0.0025, [^177^Lu]Lu-PSMA-1-DOTA vs PBS;)

Given that there was approximately 50% less [^68^ Ga]Ga-PSMA-1-DOTA in tumors than either [^68^ Ga]Ga-PSMA-11 or [^68^ Ga]Ga-PSMA I&T (Figure S6), we sought to determine if PSMA-1-DOTA would be as a suitable ligand for RLT. PSMA-1-DOTA was labeled with ^177^Lu. Typically, a crude yield of > 95% and a radiochemical purity > 94% were achieved. The identity of [^177^Lu]Lu-PSMA-1-DOTA was confirmed by Radio-HPLC and Radio-TLC (Figure S7). The efficacy studies were performed in mice bearing PC3pip tumors. As seen in Fig. 4, dosing with [^177^Lu]Lu-PSMA-1-DOTA was able to eliminate tumor growth for 24 days similarly to [^177^Lu]Lu-PSMA-617, which was used as a positive control for RLT. More dosing studies will be required to directly compare the efficacy of [^177^Lu]Lu-PSMA-1-DOTA and [^177^Lu]Lu-PSMA-617.

### Toxicity Studies in Mice

To evaluate the toxicity of PSMA-1-DOTA, a GLP extended single-dose intravenous toxicity study with PSMA-1-DOTA in NMRI mice followed by a 14-day recovery period was performed. The study was performed using a dose 1000 times the maximum human micro-dose of 100 ug and showed no mortality during the study. No significant differences were observed in the overall body weight and no abnormal clinical signs were observed during the study. There were no significant changes in clinical chemistry parameters on Day 2 or at the end of recovery period on Day 15. No macroscopic changes were observed. Therefore, the single-dose intravenous bolus administration of the test item was well tolerated in mice, however test item related histopathological changes were observed after single dosing in spleen, bone marrow and the inguinal lymph nodes. These findings were still present after 14-day recovery period, in addition lower white blood cells were observed in clinical pathology.

### “First-in-Human” PET Imaging of ^68^ Ga-PSMA-1-DOTA

**Fig. 5:**
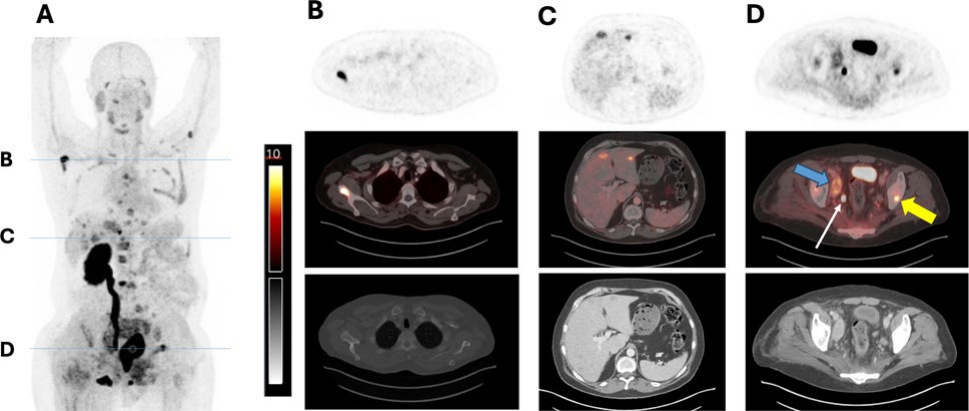
Single-patient compassionate use of [^68^ Ga]Ga-PSMA-1-DOTA. **A** Compassionate use of [^68^ Ga]Ga-PSMA-1-DOTA in a PET scan of a patient with metastatic prostate cancer who had progressed after radioligand therapy with ^177^Lu-PSMA-I&T, maximum intensity project (MIP). The patient is status post left nephrectomy for benign disease. Note the low uptake in the salivary glands, liver and spleen compared to PSMA-avid metastases. **B-D** Selected trans-axial cross-sections cut from panel **A** showing from the top: PET, PET/CT overlay, and CT at **B** metastasis in the right scapula, not osteoblastic on CT; **C** lower liver uptake and 2 liver metastases; **D** right external iliac metastasis (blue arrow) and left iliac metastases (yellow arrow) and ureter (thin arrow). The tumor to salivary uptake ratio (t/s) was: t/s = 5.63 for **B**; t/s = 2.08 for **C**; and t/s = 2.58 (blue arrow) and 2.84 (yellow arrow) for **D**. There are several other metastases throughout the body whose cross-sections are not shown

Working with Dr. Wolfgang Weber from the Technical University of Munich (TUM), a compassionate use PET imaging study was performed with [^68^ Ga]Ga-PSMA-1-DOTA on a patient with metastatic castration resistant prostate cancer who did not respond to therapies. As shown in Fig. 5, the uptake of [^68^ Ga]Ga-PSMA-1-DOTA in the salivary glands was reduced markedly with SUV_max_ of 3.9 ~ 4.4 in comparison to the published values of 11.7 ~ 17.9 from using other PSMA-radioligands [31, 32] used in man. Besides the lower uptake in the salivary glands, a rapid clearance of [^68^ Ga]Ga-PSMA-1-DOTA resulted in an over-all lower background, especially in the liver and spleen (Fig. 5A), which benefits the visualization of tiny and subtle uptake in the metastases. From Fig. 5A, we selected three trans-axial cross sections through the metastatic sites as shown in Fig. 5B-D. The tumor to salivary uptake ratio of PSMA-1-DOTA was measured for each of the sections and reported in Fig. 5B-D.

## Discussion

Xerostomia is a significant issue affecting quality of life as demonstrated in patients treated with external beam radiation therapy for head and neck cancer, encountering problems with mastication, swallowing, sleep, and speech; a burning sensation in their mouths; and dysgeusia. Some cases of xerostomia are irreversible [11]. The situation for RLT is different even though fundamental cell-specific biological outcomes may overlap. In targeted RLT, many patients who experience severe xerostomia when treated with ^225^Ac-PSMA RLT either refuse to continue or opt out of this potentially life-saving treatment due to intolerable xerostomia [2, 33]. Xerostomia and its severity depend solely on the amount of RLT targeting the salivary glands [34], different from a typical pharmacology profile, or differential PSMA expression profile among cancer patients.

Stark deterioration in the quality of life due to PSMA-targeted RLT-induced xerostomia in prostate cancer patients must be addressed to improve the therapy. Despite the unmet clinical need, the specific molecular design to reduce dose-limiting salivary gland uptake of small molecule therapeutic PSMA-targeted radioligands has not had a significant impact on salivary gland uptake. The ultimate goal of our development efforts is to prevent severe damage to the salivary glands while preserving prostate cancer targeting for imaging and therapy.

As a major step in accomplishing this goal, PSMA-1-DOTA was designed for higher binding affinity to PSMA than PSMA-11 and PSMA I&T (Fig. 2). The results of our study indicate that PSMA-1-DOTA shows promise as a novel ligand for PSMA-targeted radioligand therapy (RLT). The primary findings demonstrate that [^68^ Ga]Ga-PSMA-1-DOTA exhibits much lower uptake in the salivary glands and kidneys compared to [^68^ Ga]Ga-PSMA-11 and [^68^ Ga]Ga-PSMA I&T as observed in both mouse models (Figs. 3 & S6, Table 1), not influenced by the “tumor sink” effect (Figure S5), and a single human patient. This reduction in uptake in non-target tissues is crucial, as it suggests a lower risk of side effect toxicity, particularly xerostomia, which is a significant limitation of current PSMA-targeted therapies.

The “first-in-human” scan (Fig. 5) demonstrated a much lower [^68^ Ga]Ga-PSMA-1-DOTA uptake in the salivary glands along with an overall lower background corroborating our preclinical animal studies. From this single compassionate use patient, [^68^ Ga]Ga-PSMA-1-DOTA tumor/salivary gland uptake ratios (t/s: 5.63, 2.84, 2.58, 2.08 for different tumors, respectively, Fig. 5) were greater than the published clinically measured [^68^ Ga]Ga-PSMA-11 ratios. For 237 patients scanned with [^68^ Ga]Ga-PSMA-11, the numbers in the high (t/s > 1.5), intermediate (t/s 0.5–1.5), and low (t/s < 0.5) groups were 56 (23.6%), 163 (68.8%), and 18 (7.6%), respectively. [35] The reduced salivary gland uptake of [^68^ Ga]Ga-PSMA-1-DOTA observed in preclinical models and a single compassionate use case suggests potential for lower normal tissue toxicity in patients when PSMA-1-DOTA is labeled with alpha or beta particle emitting radionuclides, while retaining effective RLT of tumors. Future clinical investigations involving more PET imaging and therapeutic trials with [^68^ Ga]Ga-PSMA-1-DOTA and [^177^Lu]Lu-PSMA-1-DOTA, respectively, will further validate this potential.

[^177^Lu]Lu-PSMA-1-DOTA was also tested for therapeutic efficacy in a mouse tumor model (Fig. 4). Despite exhibiting lower tumor uptake, [^177^Lu]Lu-PSMA-1-DOTA demonstrated significant tumor inhibitory activity in these models. This result indicates that the lower uptake and faster clearance of PSMA-1-DOTA may not compromise its therapeutic efficacy. The observed growth of control tumors suggests that PSMA-1-DOTA, when labeled with therapeutic radionuclides, can effectively kill cancer cells, and could be a viable candidate for treatment trials, particularly for patients who experience severe xerostomia from current PSMA-targeted RLTs.

This proof-of-concept study has certain limitations. Figure 2 illustrates a radio-competition study utilizing PC3pip cells, which overexpress PSMA, showing a higher binding affinity for PSMA-1-DOTA compared to other ligands employed in PET imaging and RLT. The competition with the radiolabeled ligand, ^3^H-ZJ24, indicates that all ligands target the same site on PC3pip cells. Additional blocking experiments using cold PSMA-11 prior to [^68^ Ga]Ga-PSMA-1-DOTA injection and PET imaging may substantiate this conclusion. Scans with microPET and clinical PET using [^68^ Ga]Ga-PSMA-1-DOTA at 1 h post-injection reflected only initial uptake, not retention, even though our mouse treatment study with [^177^Lu]Lu-PSMA-1-DOTA showed efficacy (with [^177^Lu]Lu-PSMA-617 treatment as a positive control), and thus indirectly verified sufficient tumor retention. Further studies to optimize dosing of [^177^Lu]Lu-PSMA-1-DOTA may improve its therapeutic efficacy. In addition, the pharmacokinetics and biodistribution of PSMA-617 is different from that of PSMA-11 or PSMA I&T, suggesting that a direct comparison between [^68^ Ga]Ga-PSMA-1-DOTA and [^68^ Ga]Ga-PSMA-617 pharmacokinetics and biodistribution is needed. Similarly, direct comparison with similar dosing between [^177^Lu]Lu-PSMA-1-DOTA and [^177^Lu]Lu-PSMA-617 is also needed for longer term (days) biodistribution, retention, and direct efficacy comparisons. Further investigations will also entail a temporal biodistribution study along with mechanistic studies to illustrate the mechanism for the lower uptake of PSMA-1-DOTA in the salivary glands and kidneys.

It has been reported that [^225^Ac]Ac-PSMA-617 elicited a stronger response from the same patients who had limited response using [^177^Lu]Lu-PSMA-617^1^. While the VISION trial demonstrated that [^177^Lu]Lu-PSMA-617 induced xerostomia was mostly reversible in patients, severe salivary gland toxicity is more severe after [^225^Ac]Ac-PSMA-617 RLT [4]. Considering the preferable uptake profile of PSMA-1-DOTA, the next step is to start the preparations for pre-clinical studies to assess salivary and kidney uptake, damage (or lack-there-of), and tumor control using [^225^Ac]Ac-PSMA-1-DOTA. A key to this assessment is to measure the extent of dry mouth in mice during and after [^225^Ac]Ac-PSMA-1-DOTA therapy, which will require quantification of induced salivation [36], since certain degrees of xerostomia observed clinically is not prominently reflected in histopathological features.

There is ongoing debate over whether radioligand uptake by mouse salivary glands serves as a reliable predictive model for salivary gland uptake of RLTs in humans. Clearly interpretation of uptake of [^68^ Ga]Ga-PSMA-1-DOTA into salivary glands in a single human patient is not statistically significant and therefore limited, even though it correlates with data obtained in mice. Although more patient scans are desirable, the data from this study demonstrated that the lower uptake of PSMA-1-DOTA in the salivary glands happened in both mice and a human (single patient) despite inter-species differences encouraging further assessment of the clinical utilities of PSMA-1-DOTA as an RLT.

## Conclusion

In summary, the favorable uptake profile and effective tumor inhibition observed with PSMA-1-DOTA highlight its potential as a safer and effective alternative to current PSMA-targeted radioligands. Future clinical trials are warranted to confirm these findings and to establish optimal dosing protocols for the use of PSMA-1-DOTA in targeted α-therapy and other radioligand treatments.

## Supplementary Information

Below is the link to the electronic supplementary material.

Supplementary file1 (PDF 1137 KB)

## Data Availability

The reconstructed images and assay data are available upon request.
