# Supplementary material for: PSMA-1-DOTA Potentially for Effective Targeted Radioligand Therapy of Prostate Cancer
Source: Mol Imaging Biol. 2025 Sep 2;27(5):785–96. doi: 10.1007/s11307-025-02046-9 (PMC12628472; doi:10.1007/s11307-025-02046-9)
Supplement: Supplementary file 1 — Supplementary file1 (PDF 1137 KB) [file 11307_2025_2046_MOESM1_ESM.pdf]

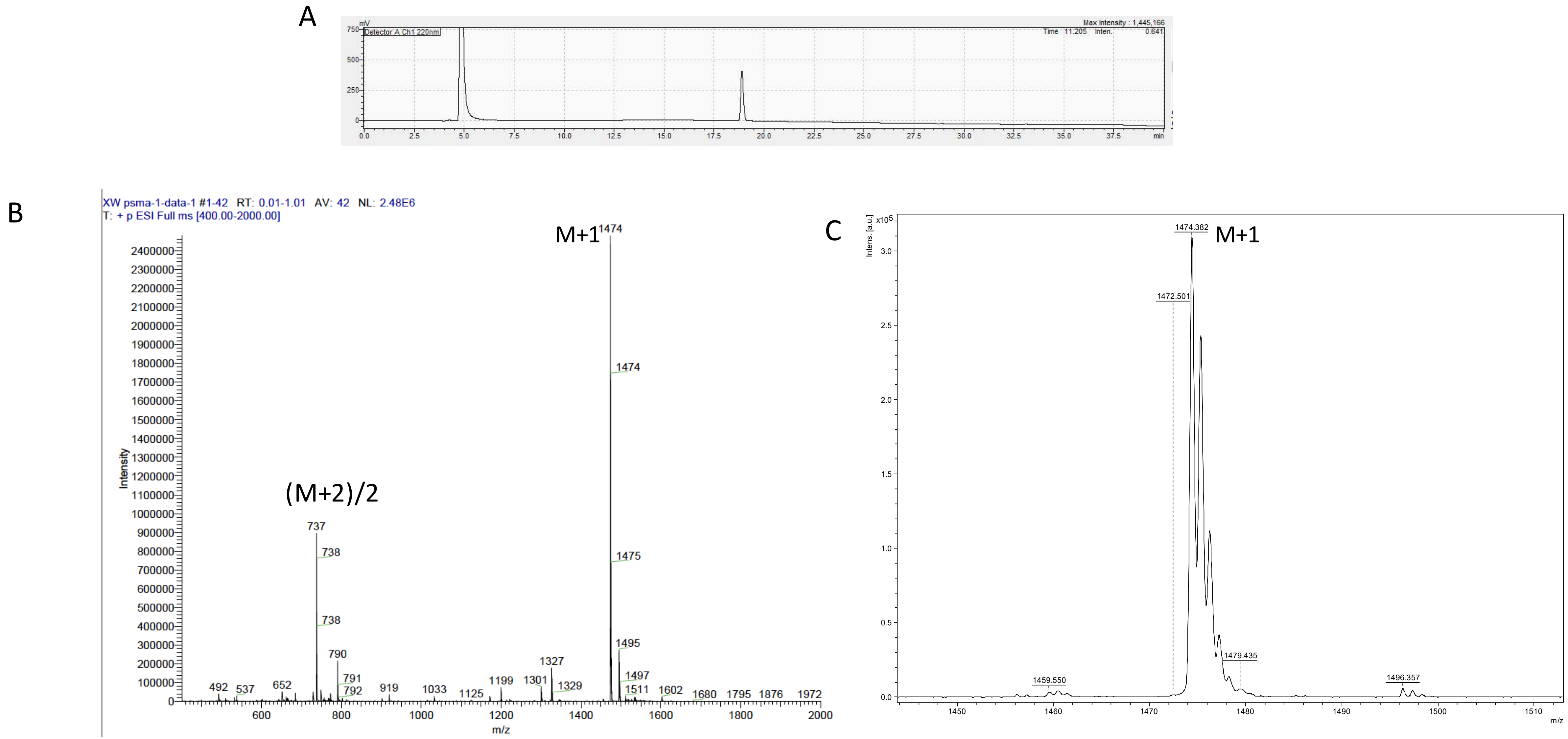

Figure S1: HPLC (A), ESI-MS (B) and MALDI-TOF-MS spectrum (C) of PSMA-1-DOTA. Calculated m/z of PSMA-1-DOTA is 1474.69.

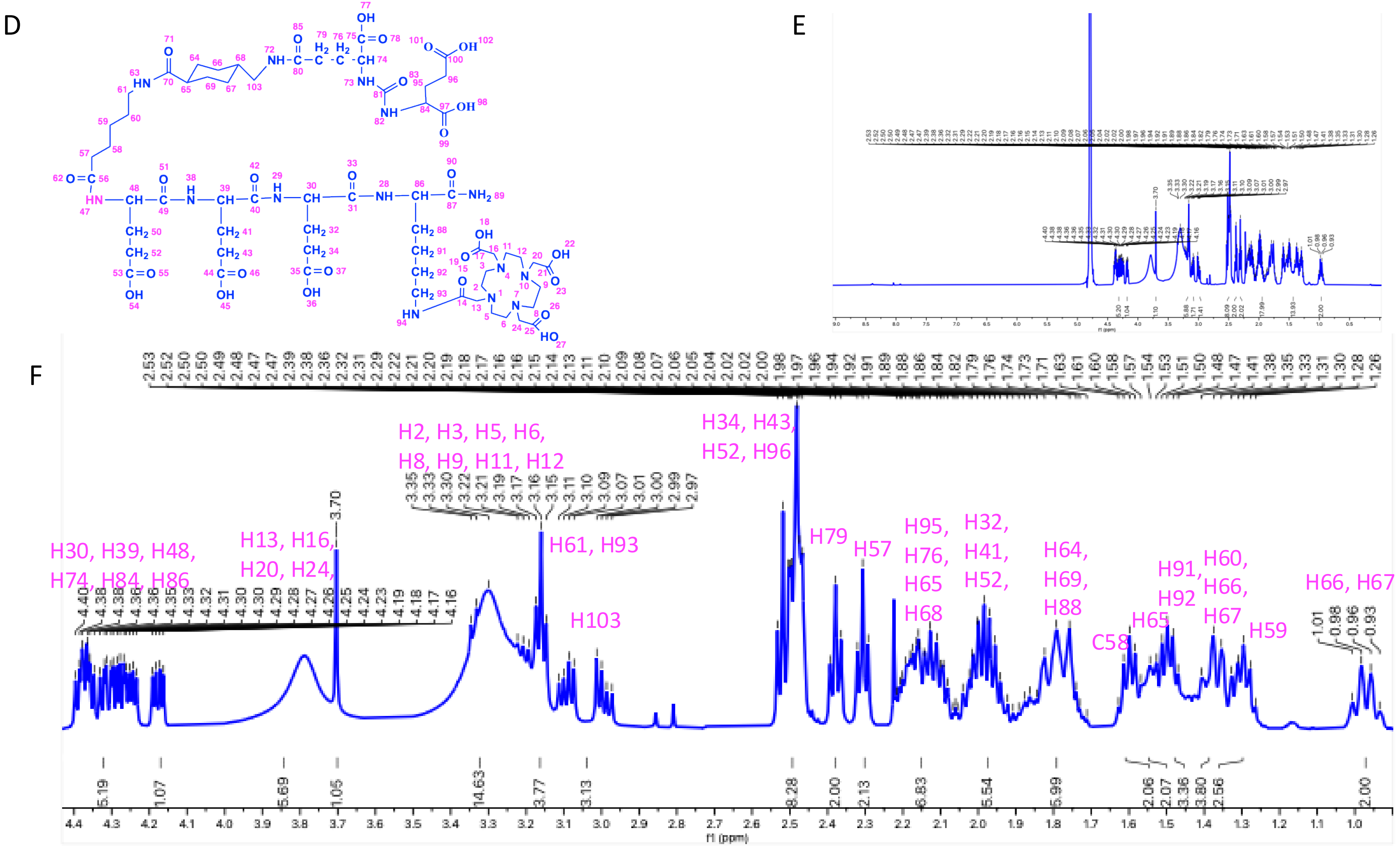

Figure S1 (cont.): ^{1}H NMR of PSMA-1-DOTA in D_2O. (D). Numbered structure of PSMA-1-DOTA. (E). Full ^{1}H NMR spectrum of PSMA-1-DOTA in D_2O. (F) Zoomed ^{1}H NMR of PSMA-1-DOTA in D_2O.

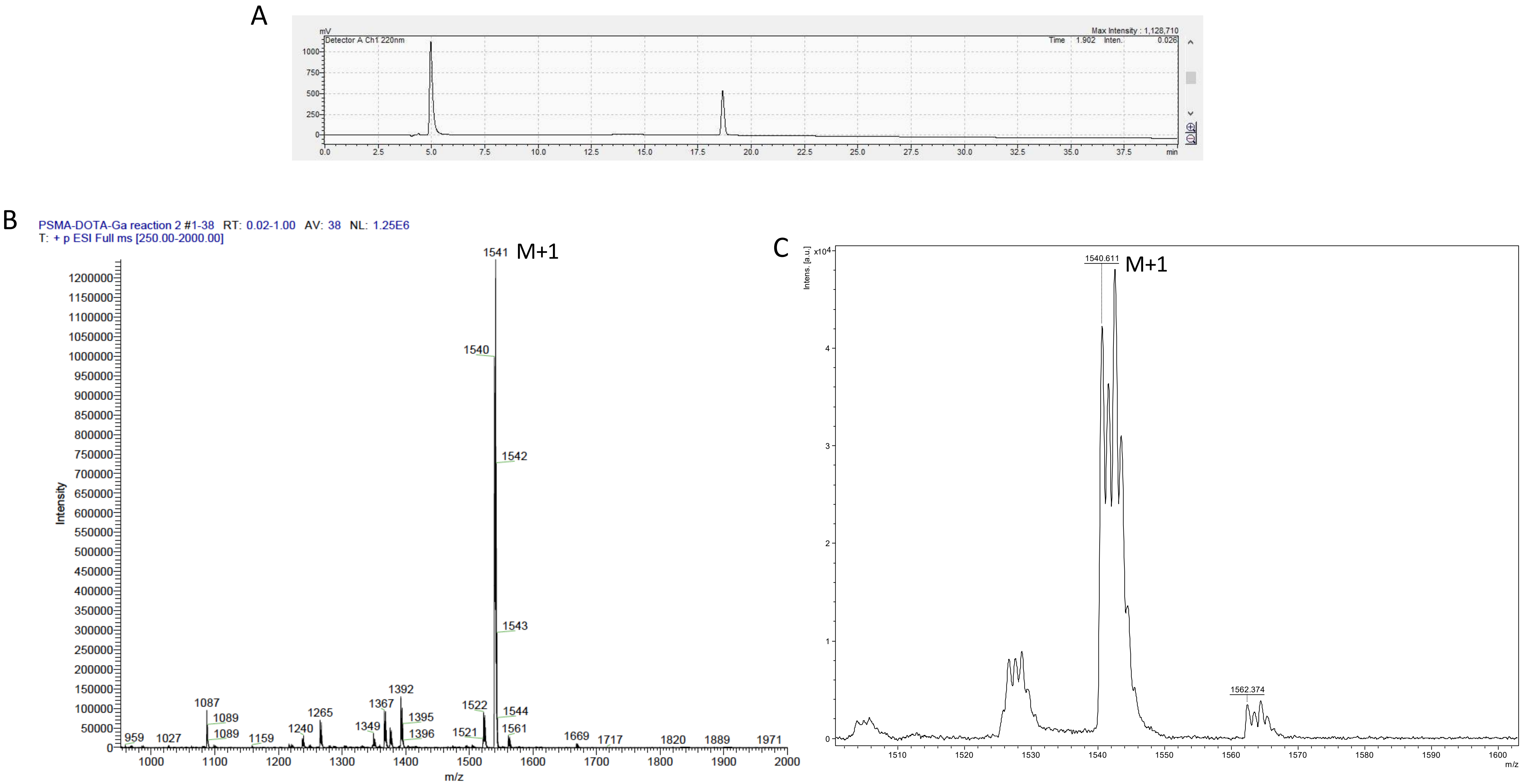

Figure S2: HPLC (A), ESI-MS (B) and MALDI-TOF-MS spectrum (C) of Ga-PSMA-1-DOTA. Calculated m/z of PSMA-1-DOTA is 1540.60.

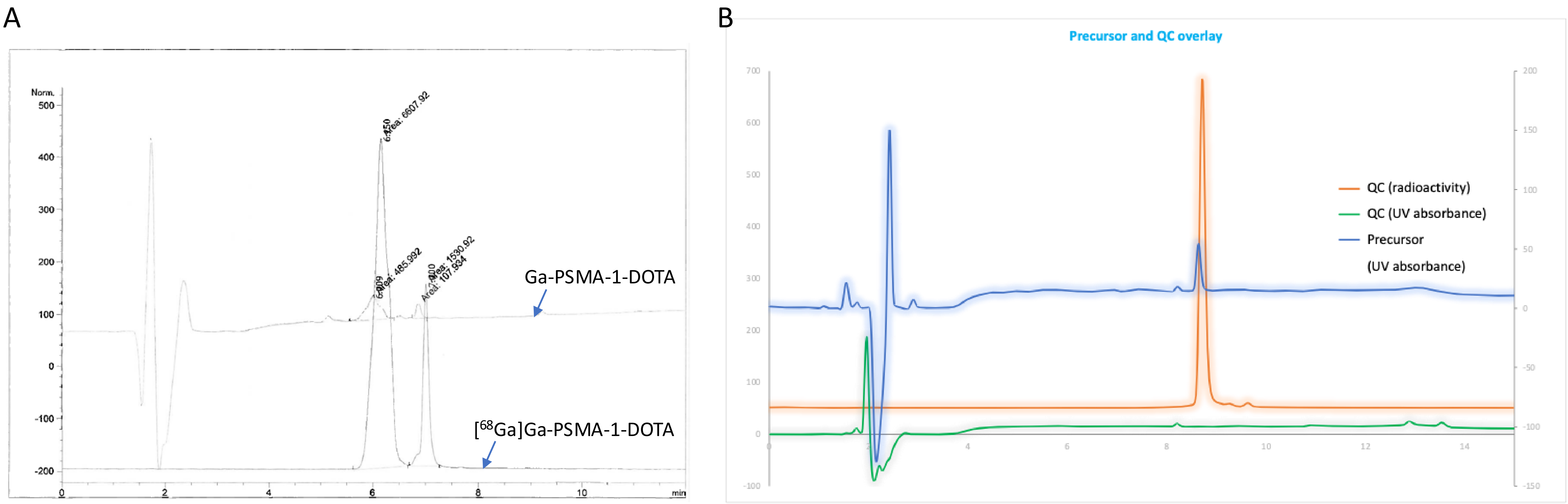

Figure S3: Characterization of ^{68}Ga-labeling of PSMA-1-DOTA. (**A**) HPLC of Radio-labeled of PSMA-1-DOTA spiked with Ga-PSMA-1-DOTA showing an overlay of [^{68}Ga]Ga-PSMA-1-DOTA (radiation detector) with cold standard, Ga-PSMA-1-DOTA (UV detector). This was the first try. Both the cold standard and radiolabeled showed two peaks at 6.1 min and 7.0 min, respectively, which might be due to diastereomers. Gradient used was 100% of 0.1% TFA in water to 100% of 0.1% TFA in acetonitrile in 12 minutes. We switched the vendor of trans-4-(Fmoc-aminomethyl)cyclohexanecarboxylic acid, re-synthesized PSMA-1-DOTA, radiolabeled it with ^{68}Ga and achieved a single peak as shown in (**B**). (**B**) Labeling of PSMA-1-DOTA showing overlay of HPLCs for [^{68}Ga]Ga-PSMA-1-DOTA (radiation or UV detector) with precursor PSMA-1-DOTA (UV detection). Gradient used was 100% of 0.1% TFA in water to 100% of 0.1% TFA in acetonitrile in 15 minutes.

C Chromatogram: ^{68}Ga

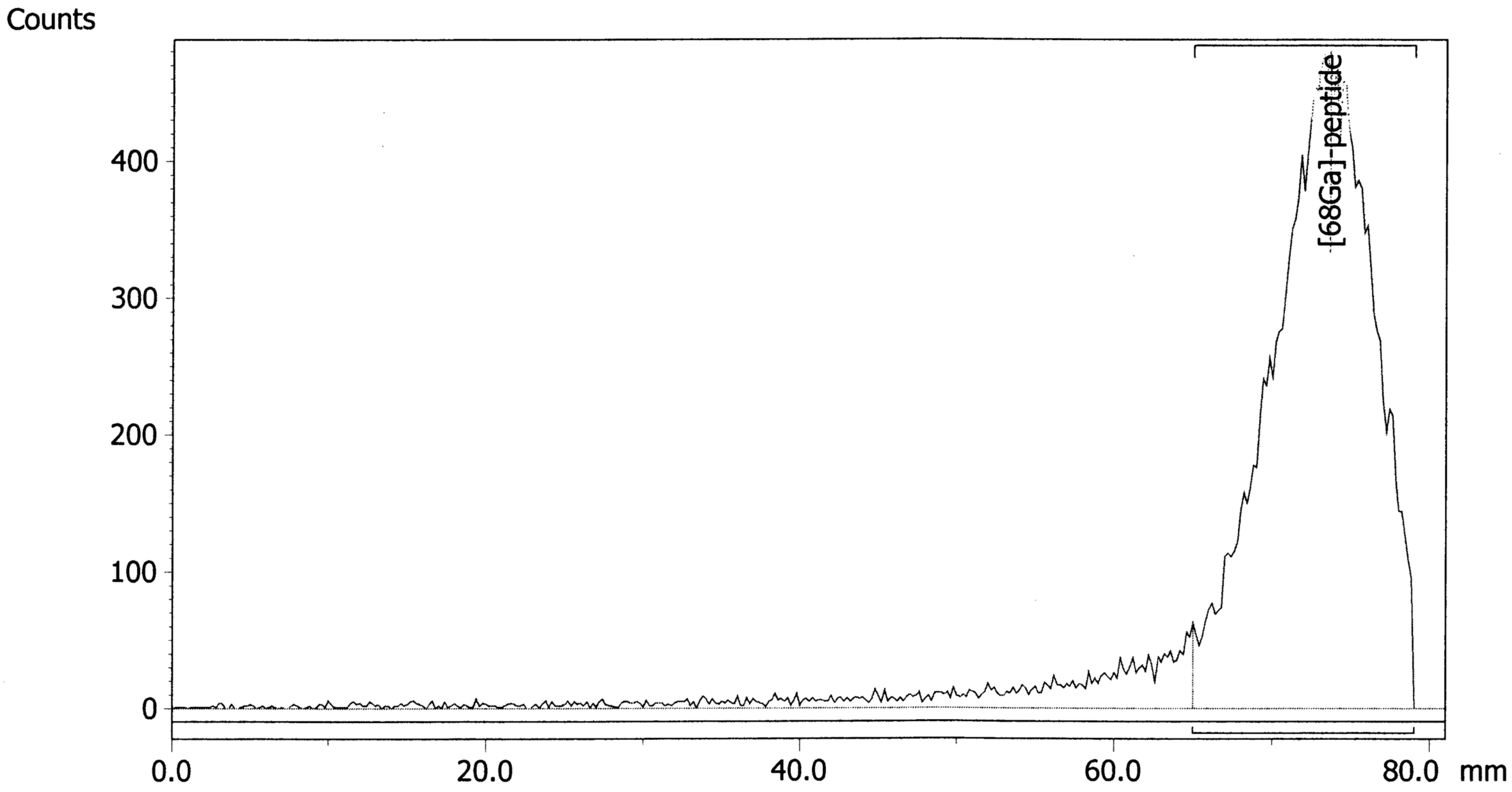

Figure S3 (cont.): (C) Radio-TLC of [^{68}Ga]Ga-PSMA-1-DOTA.

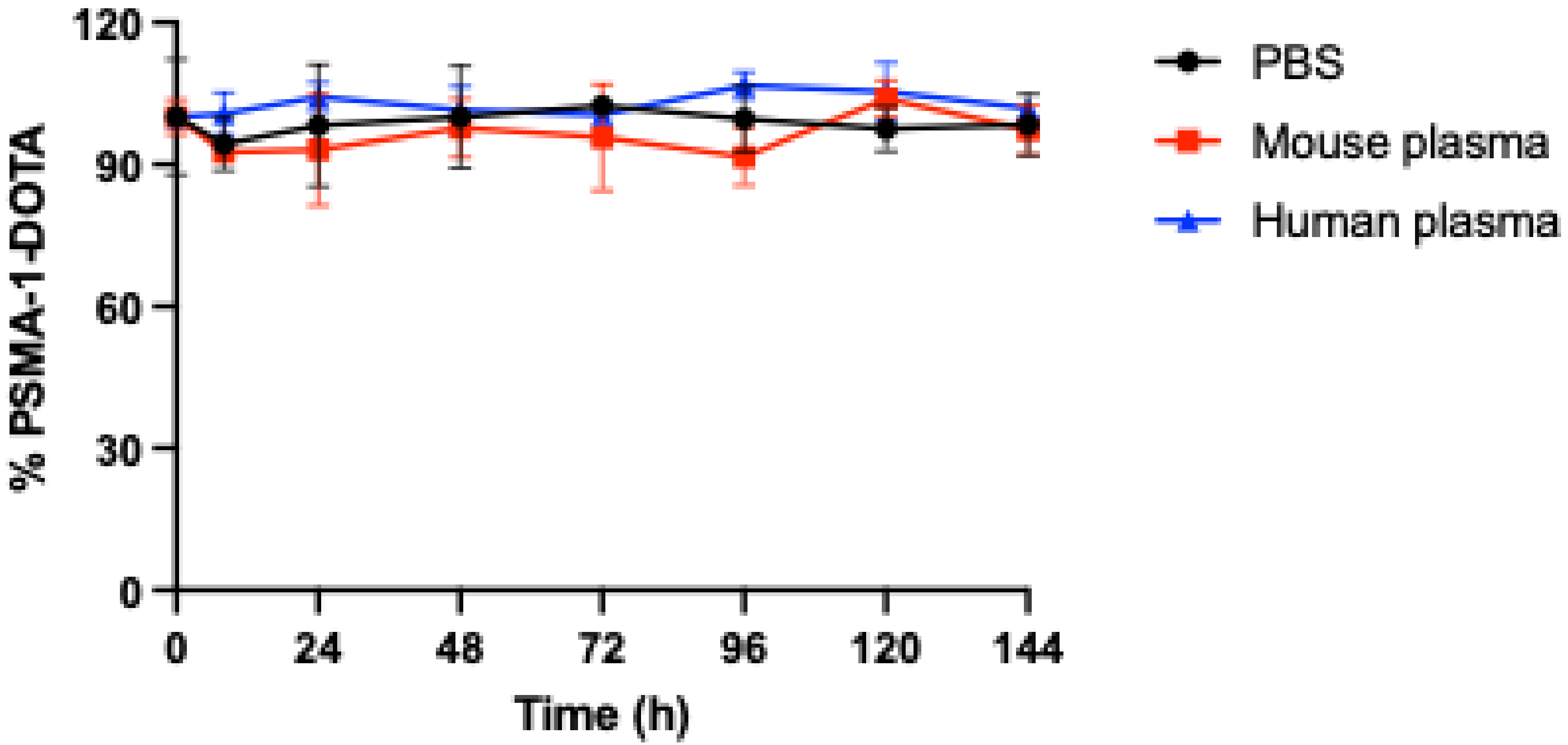

Figure S4: Stability of PSMA-1-DOTA in PBS, mouse plasma and human plasma at 37°C. Values are mean± SD of triplicates. Percentage of PSMA-1-DOTA was determined by comparing quantitative HPLC at each time point to the starting material.

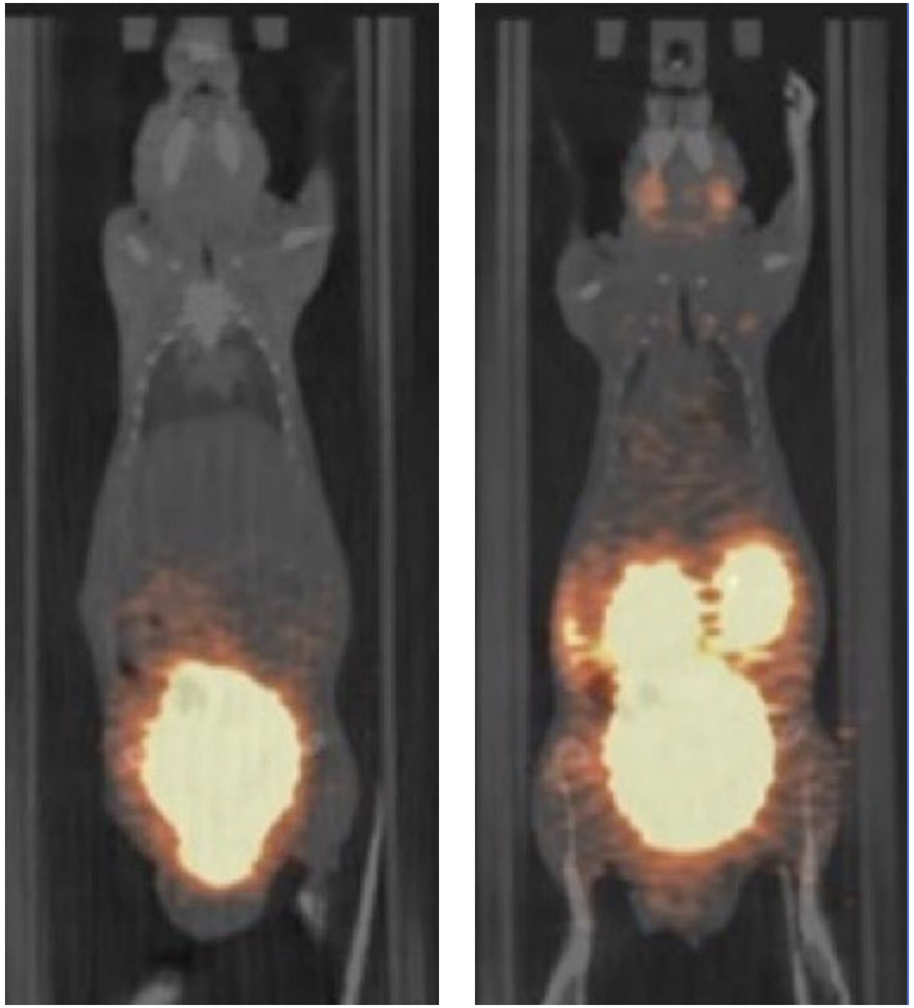

[68Ga]Ga-PSMA-1-DOTA **[68Ga]Ga-PSMA-11**

Figure S5. [68Ga]Ga-PSMA-1-DOTA/PSMA-11 microPET comparison of uptake/retention of RLT in salivary glands in animals *without* tumor. Comparison performed in same animals (n=3) doses 3.7-7.5 MBq (100-200 uCi) separated by 24 hours. [68Ga]Ga-PSMA-1-DOTA is not detectable in salivary glands and has little kidney uptake/retention, while [68Ga]Ga-PSMA-11 has significant salivary and kidney uptake/retention. Data is normalized and displayed on the same scale.

A

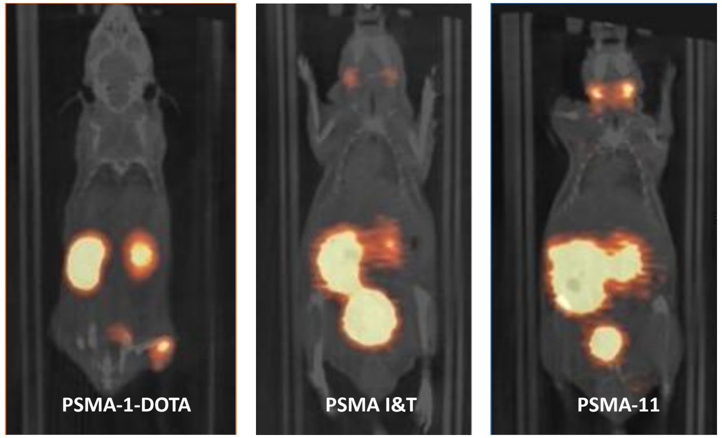

B

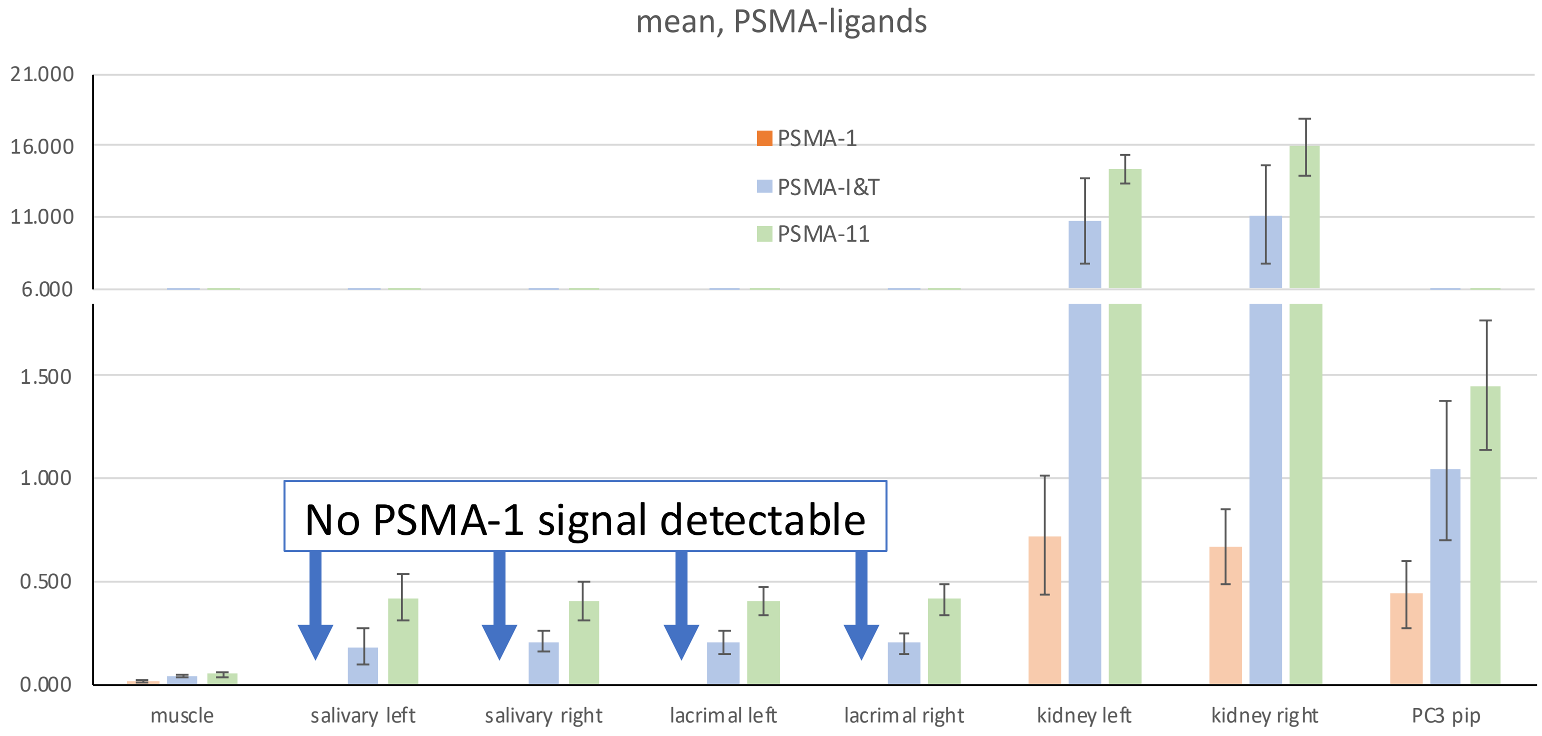

Figure S6: Comparison of [^{68}Ga]Ga-PSMA-1-DOTA, [^{68}Ga]Ga-PSMA-11 and [^{68}Ga]Ga-PSMA I&T.
(A) Overlay of microPET/CT images. (B) Quantification of micro PET/CT images at 1 hour post injection. (N=3)

A

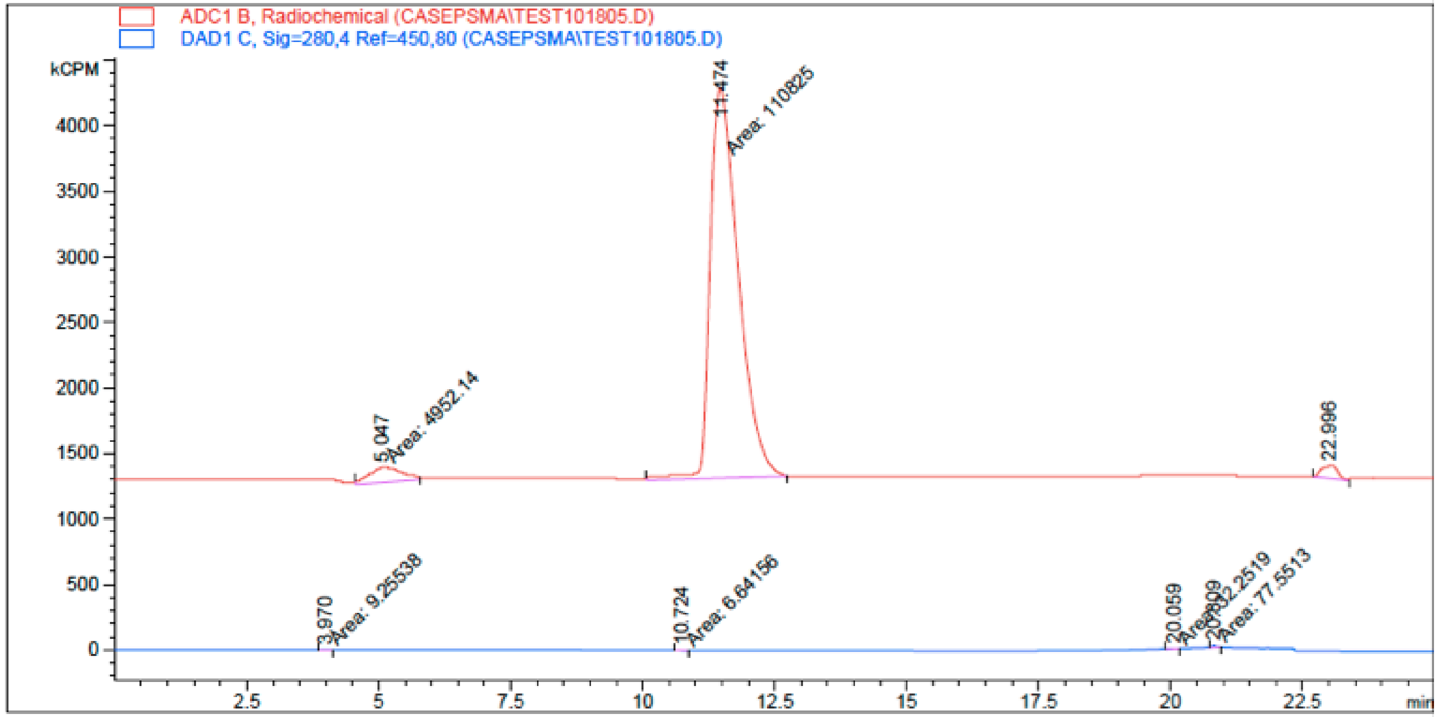

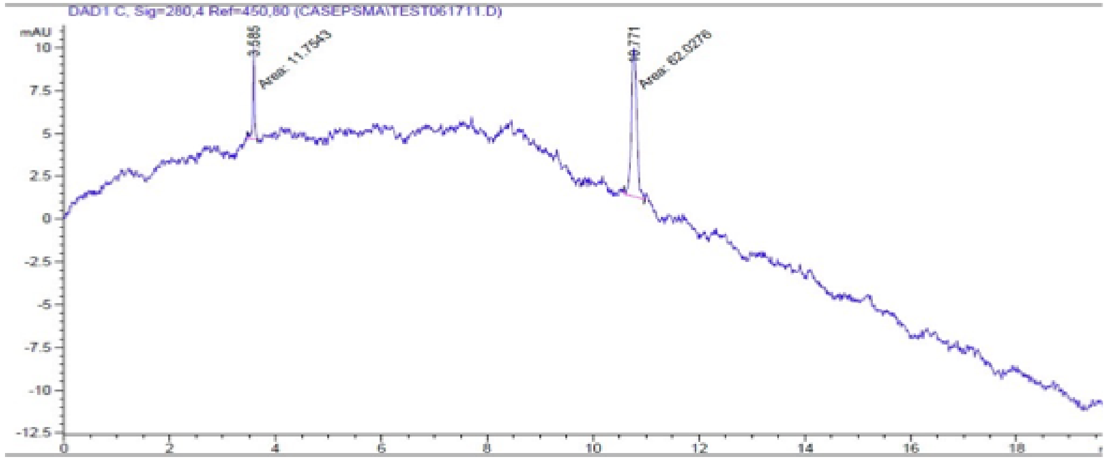

Figure S7: Identity of [^{177}Lu]Lu-PSMA-1-DOTA. (A) Radio-HPLC of [^{177}Lu]Lu-PSMA-1-DOTA. The UV chromatogram is scaled (bottom) to show the peaks, but with electrical noise/drift.

B

Instrument Parameters

Method:	CasePSMA	File:	22101903.R001
Evaluated:	19 Oct 2022 15:49:30	Created:	19 Oct 2022 15:49:30
Evaluation by:	PAT		
Collimator Type:	Hi Efficiency	Width:	10 mm
Elect. Resol:	Normal	Amp. Range:	50 - 2047
Resolution:	256 chan	Chan Size:	0.844 mm
Hi Voltage:	1449 Volts	Chan of Zero mm:	7.9
Run Time:	1.00 min		
Relative Pos:	0.0 mm		

Comments

Method for use in the analysis of [Lu-177]Lu-PSMA-1-DOTA
3 hours @ 90C

Analysis Parameters

Bkg Subtraction:	none	Origin:	0.0 mm
Normalization:	none	Front:	200.0 mm
Total Counts:	74525.0 (74525.0 CPM)	Region:	0.0 - 200.0 mm
Total File Counts:	74637		

Region Analysis

Definition:	Peak Search
Peak Slope:	2.0 counts/mm
Min Width:	2.0 mm
Min pct of Total:	0.0 %

Reg	(mm) Start	(mm) Stop	(mm) Centroid	RF	Region Counts	Region CPM	% of Total	% of ROI
Rgn 1	11.1	28.8	19.7	0.098	70780.0	70780.0	94.97	99.77
Bkg 1	45.7	55.8	50.2	0.251				
Rgn 2	63.4	71.8	67.1	0.336	161.2	161.2	0.22	0.23
Bkg 2	79.4	98.8	87.6	0.438				
2 Peaks					70941.2	70941.2	95.19	100.00

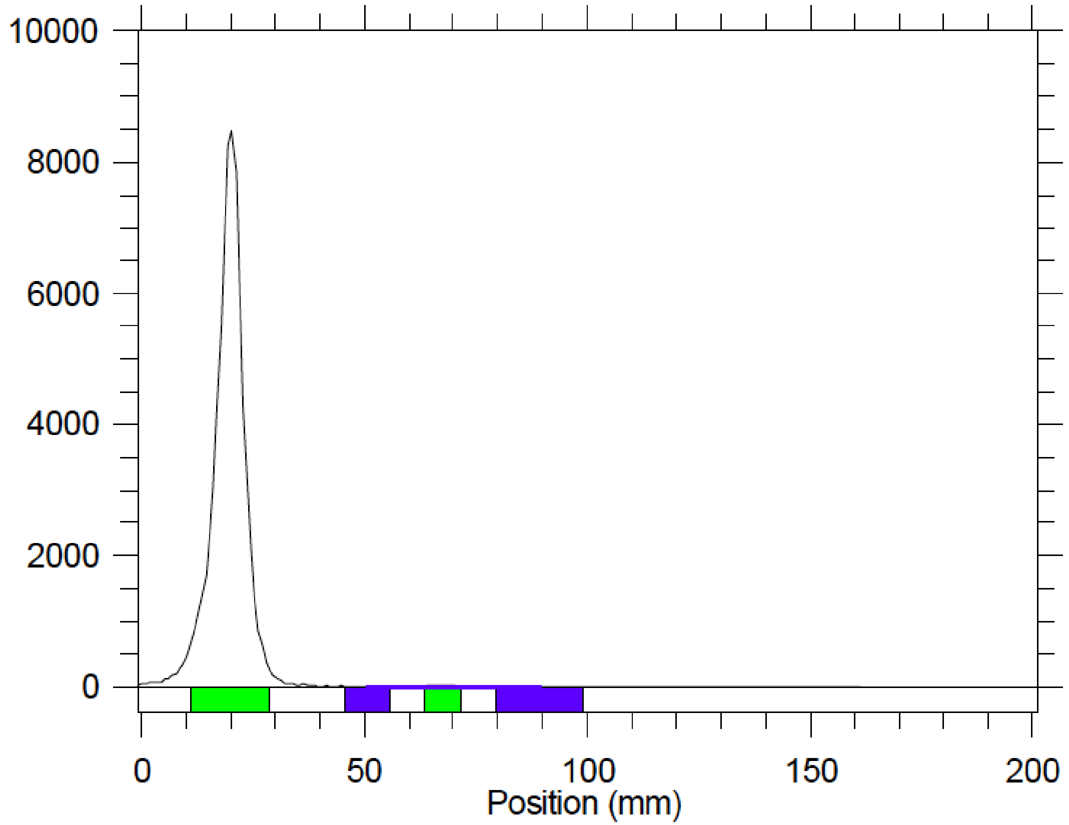

Figure S7 (cont.): Identity of [^{177}Lu]Lu-PSMA-1-DOTA. (B) Radio-TLC of [^{177}Lu]Lu-PSMA-1-DOTA showing mostly a pure compound.
